# Bibliometric and visualized analysis of dynamic balance and brain function using web of science and CiteSpace from 1995 to 2022

**DOI:** 10.1016/j.heliyon.2024.e24300

**Published:** 2024-01-08

**Authors:** Mengjiao Liu, Jian He, Dongwei Liu, Meijin Hou, Ye Ma

**Affiliations:** a Research Academy of Grand Health, Faculty of Sports Sciences, Ningbo University, Ningbo 315211, China; b School of Information Management and Artificial Intelligence, Zhejiang University of Finance and Economics, Hangzhou 310018, China; c National Joint Engineering Research Center of Rehabilitation Medicine Technology, Fujian University of Traditional Chinese Medicine, Fuzhou 350108, China; d Key Laboratory of Orthopaedics and Traumatology of Traditional Chinese Medicine and Rehabilitation, Fujian University of Traditional Chinese Medicine, Fujian, China

**Keywords:** Human dynamic balance, Brain functional structure, Functional connectivity, CiteSpace, Visualization analysis

## Abstract

**Purpose:**

This study aims to explore the dynamic balance of human beings and investigate the relationship between functional structure as well as functional connectivity. Through a comprehensive bibliometric and visual analysis of the research literature from 1995 to 2022, we quantitatively display the development of the dynamic balance and brain structure as well as functional connection. Our objective is to present new trends and frontiers in the study of dynamic balance and brain function through bibliometrics software, providing valuable insights for future research in this domain.

**Methods:**

The literature on dynamic balance, brain structure and functional connectivity between 1995 and 2022 was retrieved from the Web of Science database. We employed CiteSpace software to analyze various aspects, including the year of publication, journal, authors, keywords, institutions, countries, and references. Based on the analysis results, a co-reference map was generated to visually observe research hotspots and knowledge structures.

**Results:**

A total of 1533 records were retrieved during the survey period (1995–2022), with a gradually increase in the number of annual publications. Notably, the data suggests a notable increase in publications between 2020 and 2021. The number of publications increased by 20 % from 2020 to 2021. The journal “*Proceedings of the National Academy of Sciences* (PNAS)” emerged as the most prolific journal. Among the cited authors, Deco and Gustavo ranked at the top. Key research terms in this field include "neural network", "functional connectivity", "dynamic", "model" and "brain". Particularly, the keyword "neural network" exhibited the strongest growth. The analysis of keywords cluster revealed the top 10 clusters of research themes. Oxford University stood out as the most productive institution, while the United States held the greatest influence with the highest number of publications and centrality. The reference cluster analysis further demonstrated the top 10 clusters in the literature.

**Conclusion:**

Through the use of CiteSpace software, this study performed a comprehensive bibliometric and visual analysis of the Web of Science research literature on human dynamic balance and brain structural as well as functional connectivity over the past few decades. This may help researchers identify new perspectives on potential collaborators as well as collaborating institutions, hot topics, and research frontiers in the research field. The results provided an intuitive displayed overview of research trends, hotspots and frontiers in this field, facilitating a general understanding of its progression. Through unremitting efforts, it provides valuable guidance and reference for future research work.

## 1 Introduction

Dynamic balance is essential for performing daily activities, including walking, running, or navigating uneven surfaces. Impaired dynamic balance often leads to falls during gait [1]. Achieving proper dynamic balance requires the coordinated integration of sensory information and motor control. The brain plays a crucial role in maintaining dynamic balance, ensuring normal upright and smooth walking [2]. Maintaining balance is a complex process involving various regions of the brain, rather than being confined to a few specific regions [3]. Key structures such as the cerebellum, basal ganglia, thalamus, hippocampus, inferior parietal cortex, subparietal cortex, and frontal lobes are believed to play significant roles in balance skills, regardless of whether balance is trained or assessed [4]. These findings highlight the intricate interplay between these brain structures in achieving and maintaining balance.

Gait and balance disorders are prevalent manifestations observed in various neurological conditions, including Parkinson's disease (PD), atypical Parkinson's syndrome, idiopathic atmospheric hydrocephalus, cerebrovascular disease, dementia, and multiple sclerosis (MS) [5]. Dizziness, postural instability, ataxia, and falls are common in the majority of patients with multiple sclerosis (MS), significantly diminishing mobility, independence, and quality of life. These symptoms reflects the dysfunction of the functional integration of visual, somatosensory, and vestibular sensory cues to a large extent. Despite this high prevalence, the underlying brain regions associated with balance problems remain poorly defined. Most research has focused on lesions within the brain stem and cerebellum [6]. Studies have demonstrated that the motor control teat latency is longer in multiple sclerosis patients and is significantly correlated with the lesion volume of the cerebral cortex, medial frontal lobe, temporal lobe, lower frontal lobe, and tectal lobe [7]. Among disorders related to brain injury caused by head trauma, patients with a history of falls exhibit more impaired balance [8]. Patients with acquired brain injury (ABI) often experience cognitive balance impairments that may contribute to the risk of falls. The risk of falls after traumatic brain injury (TBI) associated with secondary injury is a concern [9]. These disorders are intricately linked to underlying structural and functional changes within the brain throughout the progression of the respective diseases.

Neuroimaging studies utilizing techniques such as functional magnetic resonance imaging (fMRI) [10] and electroencephalography (EEG) [11] have yielded valuable insights into the activation patterns of brain regions during tasks involving dynamic balance. The investigation of balance dysfunction and its relationship to brain structure and functional connectivity is an area of increasing research interest [12]. The exploration of brain connectivity has emerged as a promising avenue for understanding the pathophysiology of neurological and psychiatric disorders, providing valuable insights into the underlying mechanisms associated with gait and balance disorders [13]. Moreover, neurorehabilitation interventions show promise in promoting brain plasticity and ameliorating gait and balance impairments in affected patients, thereby improving their functional outcomes [14].

At present, there are a lot of relevant research in this field, and the simple retrieval method can no longer accurately and quickly retrieve and integrate the information needed for related research. Therefore, a more efficient, scientific, accurate and effective method is needed for retrieval and analysis. In this study, we conducted a comprehensive analysis of the literature pertaining to dynamic balance and brain function. We employed various bibliometric methods, including author collaboration networks, co-occurrence keywords, countries, institutions, co-citation citations, and co-citation outbreaks, utilizing the visual analysis tool CiteSpace [15], Through a retrospective examination of published literature between 1995 and 2022 from the Web of Science core collection, our aim is to provide a comprehensive and panoramic overview of studies on the dynamic balance of the human body and the functional structure as well as connectivity of the brain. By doing so, we aim to identify potential emerging trends and research directions, providing valuable references for future research endeavors.

## 2 Material and methods

To ensure the comprehensiveness, accuracy and high credibility of the original data, careful consideration was given to the selection of data sources. The study specifically chose Web of Science, renowned as the world's largest academic journal database, was chosen for this study. A systematic search was conducted using relevant terms, including "dynamic stability" or "dynamic balance", in conjunction with subject headings such as "brain structure" or "brain functional connectivity" or "brain network".

The comprehensive search used high-level search terms: TS = ((dynamic stability) OR (dynamic balance)) AND TS = （(brain structure) OR (brain functional connectivity) OR (brain network)). The retrieval period spans from January 1995 to December 2022, with the search language is limited to English. This search strategy yielded a total of 2705 records. Subsequently, a screening process was implemented to exclude papers that involving animals and those did not meet the research requirements. After screening, 1533 articles were included in this study. Article documents were exported for further analysis using the visualization tool, CiteSpace. We utilized CiteSpace (V6.1.R6, 64 bits), a powerful bibliometric analysis tool, to conduct our study. CiteSpace is a Java-based application specifically designed for analyzing and visualizing citation networks [16]. It offers a wide range of bibliometric research capabilities, including institutional co-citation analysis, author collaborative network analysis, and topic and domain co-occurrence visualization.

Among the 1533 articles analyzed, various types of literature were identified, including articles (1278), reviews (179), and conference proceedings (26), etc. These articles were published in a total of 835 journals, contributing to the literature on dynamic balance in the human and the structural and functional connectivity of the brain.

Using CiteSpace, we determined the timing, frequency, and centrality of the co-occurrence network, which includes annual publications, journals, countries, institutions, authors, references, and keywords. The tool facilitates visual exploration and analysis of domain structures, dynamic patterns, and emerging trends, enabling researchers to intuitively identify the evolution path of subject frontier and seminal literature.

Visualization knowledge maps depict networks in the form of common node-and-link diagrams. Nodes within various networks can represent different elements, such as authors, keywords, institutions, countries and references. In the co-occurrence plot, circles represent nodes, with the size of each node indicating the frequency of keyword occurrence, and different colors indicating different years (warmer color for more recent year color). Links between nodes represent cooperative, co-occurrence, or co-citation relationships, with larger nodes indicating higher frequency. The lines connecting nodes represent co-occurrence relationships, with the thickness of the lines indicating the intensity of co-occurrence. The purple circle represents centrality, with nodes with a high degree of centrality (>0.1) considered as pivot points or turning points in a particular domain. The version of this software is constantly being updated.

The parameters of CiteSpace used for the analysis were as follows: time slice (1995–2022), number of years per slice (3), term source (all selected), node type (selected one at a time), selection criteria (first 50 objects), pruning (pruning the network of slices) and visualization (cluster view - static).

For the analysis, the retrieved articles were saved as plain text files, including both the records and citations, and were subsequently converted to an executable format for visualization using the "Data/Import/Export" function of CiteSpace. We used CiteSpace to identify the time, frequencies, and centralities of the cooccurrence networks. We focused on a time span from 1995 to 2022, divided into three time segments and performed a comprehensive analysis, exploring various dimensions of the literature. Then each node of the figure is adjusted to accentuated connections, resulting in a visually compelling map of scientific knowledge. It includes author networks, keyword networks, clustering networks, national networks, institutional networks, and the co-citation relationships among the selected articles.

## 3 Results

We conducted a comprehensive analysis of the literature on dynamic balance and brain function. The data was visualized and key findings included the annual publication volume and top 10 journals were identified firstly. Using the visual analysis tool CiteSpace, we adopted a variety of bibliometric methods, including author cooperation network, keyword co-occurrence and cluster analysis, institution, country, co-cited citations and co-cited outbreaks.

### 3.1 Annual publication volume and productive journals

**Fig. 1:**
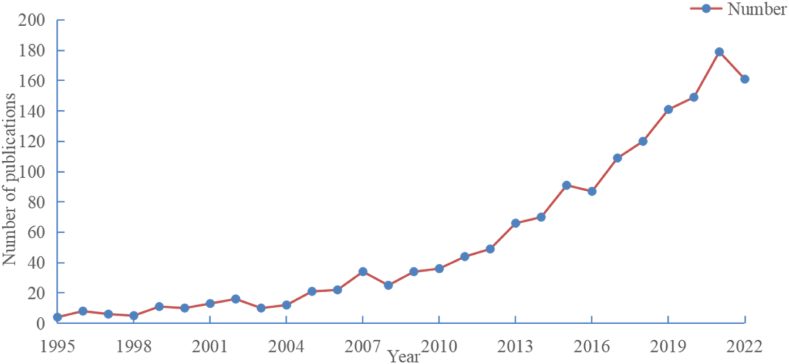
Statistical line chart of the number of literatures from 1995 to 2022.

A total of 1533 records were included in the analysis. Fig. 1 displays the annual distribution of publications, we can see the number of publications increased with some fluctuations. The graph reveals fluctuations but an overall increasing trend over the past 27 years. As the years continue to grow, especially since 2000, with the technological innovation of scientific research methods and the continuous evolution of publications, the number of publications fluctuates, and generally shows a growing trend. In 1995, only 4 publications were recorded. Subsequently, the number of publications varied from year to year, with a local peak of 34 in 2007, a drop to 25 in 2008, and a subsequent rise. The highest number of publications, 179, was observed in 2021, followed by a slight decline to 161 in 2022. This trend highlights a positive correlation between the year of publication and the number of research papers.

### 3.2 Analysis of productive journals

Table 1 provides an overview of the top 10 most productive journals in the field. Notably, *Proceedings of the National Academy of Sciences* (PNAS) ranked first with 1053publications, covering a wide range of fields including biology, physics, mathematics and social sciences. Followed by *Journal of Neuroscience* with 993 publications, focusing on medical and neuroscience-related topics. *Nature* ranked third with 854 publications, known for is publishing cutting-edge research across various scientific disciplines, particularly in neuroscience. These journals have a relatively high impact factor, averaging at 25.3524.

**Table 1:** The top 10 most productive journals.

Rank	Journals	Impact Factor	Published papers
1	Proceedings of the National Academy of Sciences (PNAS)	12.779	1053
2	Journal of Neuroscience	5.3	993
3	Nature	69.504	854
4	Science	63.714	853
5	Neuron	18.688	789
6	Nature Reviews Neuroscience	38.755	681
7	NeuroImage	7.400	678
8	Plos One	3.752	677
9	Nature Neuroscience	28.771	654
10	Cerebral Cortex	4.861	645

### 3.3 Author cooperation network analysis

Table 2 presents the top 8 authors in the collaborative network based on published literature related to human dynamic balance and brain functional structure and connectivity. Deco and Gustavo [[17], [18], [19], [20], [21]] emerged as the most prolific authors with a total of 17 published articles. Following them are Robinson [22], Breakspear et al. [23], Jiang et al. [24], Zhou et al. [25], and Dimitri et al. [26].

**Table 2:** The top 5 authors of the collaborative network.

Rank	Frequency	Centrality	Year	Authors
1	17	0.01	2011	Deco, Gustavo
2	11	0	2007	Robinson, P A
3	10	0	2006	Breakspear, Michael
4	7	0	2019	Wang, Jiang
5	7	0	2019	Zhou, Changsong
6	7	0	2020	Van de ville, Dimitri
7	6	0	2007	Jirsa, Viktor K
8	6	0	2014	Leech, Robert

**Fig. 2:**
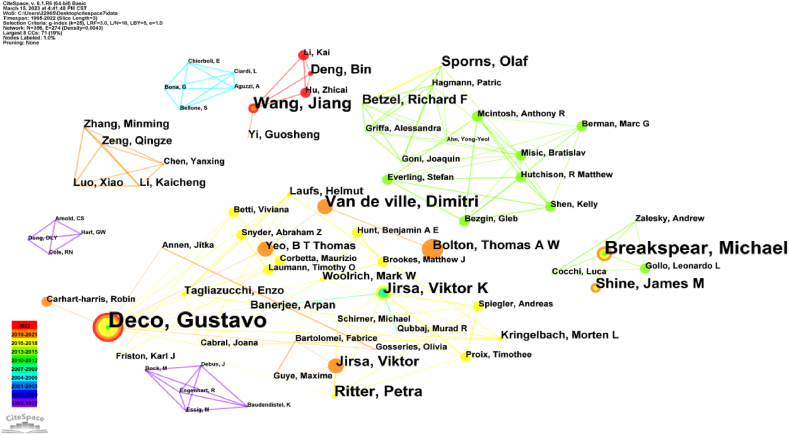
Author cooperative network map. The co-cited authors are the authors cited in the references, with only the first and second authors considered for analysis. The size and color of each circle represent the year of the author's publication, indicated by the color band in the lower-left corner corresponding to the year. Collaboration between authors is represented by linked chains. Color should be used for any figures in print.

An analysis of the author-collaboration network based on 1533 papers yielded 356 nodes and 274 links (see Fig. 2), indicating that these 1533 articles were authored by 356 authors. Among these authors, collaborative groups were observed, as well as individual authors with no strong collaborative ties. Fig. 2 represents the author collaboration network analysis of literature investigating the relationship between human dynamic balance and brain function. It provides information on the importance indicators and network attributes of each author in the network. The figure depicts the first eight cooperative networks, representing influential research groups in the field. This diagram also reflects the social relationship between authors, showing that many authors tend to collaborate within relatively stable partner groups, often two or more core authors. Notably, authors like Gustavo, and Michael, exhibit strong centrality and prominence within their respective groups.

In 2011, Deco and Gustavo published a paper on the dynamic balance of the brain at rest [17], which has been cited 207 times. The results demonstrate that brain activity at the large-scale nervous system level is not noise, but rather exhibits organized functional networks that maintain consistency over time. These networks, known as resting state networks (RSNS), are closely related to underlying anatomical connectivity. Another related paper was published in 2016 on functional dynamic connectivity in patients with major depressive disorder [27], receiving 122 citations. This paper highlights of resting state functional magnetic resonance imaging (RS-fMRI) as an effective tool to study the connectivity structure of mental health disorders.

In 2014, Zalesky and Andrew [28] published an influential article on time-resolved resting state brain networks, which has been cited 475 times. Their findings suggest that over time, changes in brain dynamics leads to alterations in the properties of complex networks, striking a balance between efficient information processing and metabolic consumption.

### 3.4 Co-occurring keywords analysis

**Fig. 3:**
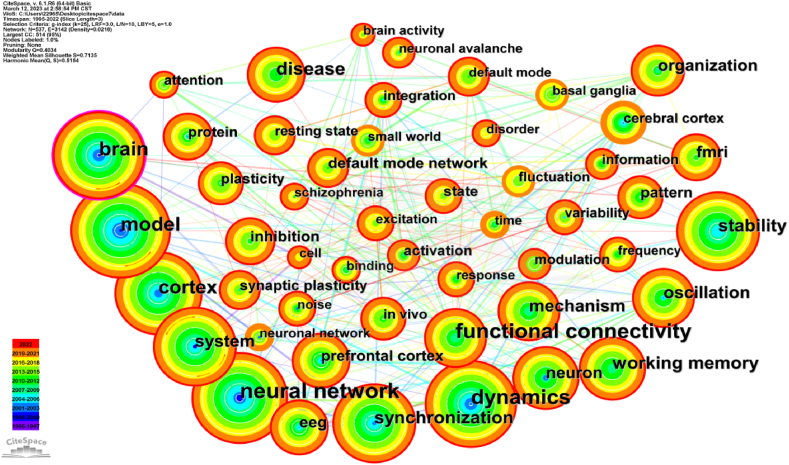
Co-occurrence map of keywords. The color of each node corresponds to the year of the first co-occurrence, as depicted by the ribbon denoting the year in the lower left corner. The color of co-occurrence links between keywords reflects the chronological order, with light purple indicating the oldest co-occurrence and red indicating the most recent co-occurrence. Nodes marked with dark purple circles typically indicate the importance and significance of these keywords. Color should be used for any figures in print.

Fig. 3 illustrates the co-occurrence of keywords, indicating research hot topics in the study of human dynamic balance and brain structure and functional connectivity. The keyword network graph composed of 537 nodes and 3142 links (see Fig. 3), revealing prominent research areas within this field. The top 50 frequently cited or mentioned keywords were identified and Table 3 presents the top 22 most cited keywords.

**Table 3:** The top 22 most frequently used keywords.

Rank	Frequency	Centrality	Year	Keywords
1	381	0.06	1995	neural network
2	330	0.04	2001	functional connectivity
3	306	0.05	1998	dynamics
4	223	0.07	1997	model
5	201	0.12	1996	brain
6	183	0.07	1995	cortex
7	139	0.06	2001	disease
8	124	0.04	2001	working memory
9	115	0.07	1998	synchronization
10	111	0.05	2002	stability
11	101	0.03	2001	oscillation
12	98	0.06	1997	system
13	93	0.03	2005	mechanism
14	87	0.02	2007	organization
15	78	0.04	2001	neuron
16	74	0.03	2008	fMRI^a^
17	72	0.03	2001	EEG^b^
18	55	0.03	2010	default mode network
19	51	0.05	2001	prefrontal cortex
20	51	0.02	2007	pattern
21	51	0.01	2010	inhibition
22	47	0.04	2007	plasticity

a fMRI, functional Magnetic Resonance Imaging; an emerging method of neuroimaging.

b EEG, electroencephalogram; a medical test that measures and records electrical activity in the brain.

Fig. 3 shows the 22 most frequently used keywords. Among the selected keywords, "Neural network" emerges as the most frequently used keywords, cited 381 times, followed by "functional connectivity" (330 times), "dynamics" (306 times) and "model" (223 times). Other popular keywords include "brain" (201 times), " cortex" (183 times), "disease" (139 times) and "working memory" (124 times). For instance, "brain" exhibits the highest centrality value of 0.12, followed by "cortex" (0.07), "model" (0.07), and "synchronization" (0.07). Consequently, extensive research has been conducted on the brain [29] and cortex [30] within this field.

Moreover, investigations have explored the relationship between brain structure and neurodegenerative diseases, particularly those associated with gait disorders. Parkinson's disease, for example, is characterized by a prolonged course and requires considerable recover time. Modern medical techniques, such as functional magnetic resonance imaging (fMRI) [31], an emerging neuroimaging method, and electroencephalography (EEG) [11], a medical test that measures and records electrical activity in the brain, have been employed in studying these conditions.

**Fig. 4:**
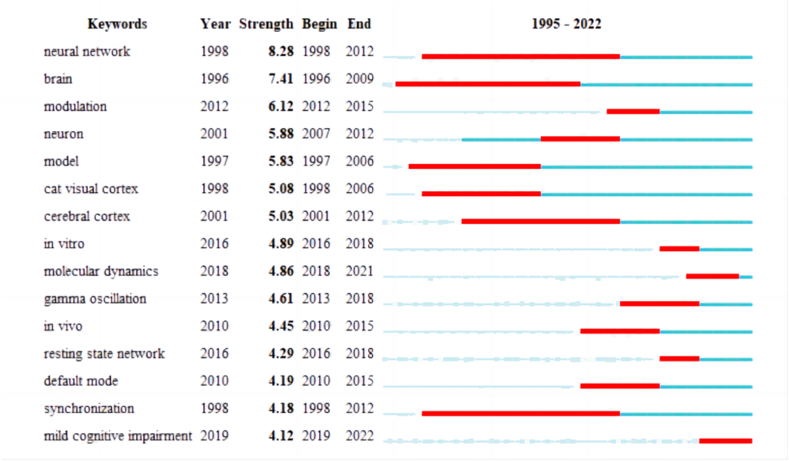
Top 15 Keywords with the Strongest Citation Bursts. All these keywords exhibit significant explosive intensity; they are sorted based on the time the outbreak began, and the start to end times are indicated by red terms. Color should be used for any figures in print.

Fig. 4 displays the top 15 keywords with strong explosive intensity from 1995 to 2022 Among them, "mild cognitive impairment" is the targeted intervention for brain balance. The keyword "neural network" which appeared in 1998, had the highest explosive intensity, reaching 8.28. Similarly, "brain" experienced a surge in citations between 1996 and 2009, with an explosive intensity of 7.41, while "modulation" experienced a surge between 2012 and 2015, with the intensity of 6.12. Keywords such as "cerebral cortex", "neuron", "resting state network" and "molecular dynamics" reflect the research directions of brain structure and network.

**Fig. 5:**
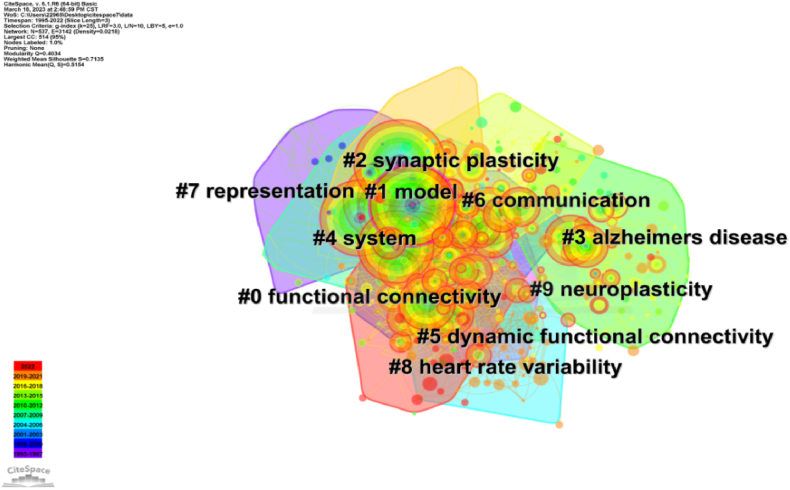
Cluster map of Keywords. The figure showcases ten clusters labeled from #0 to #9, with the sequence number indicating cluster size. Cluster #0, "functional connectivity," is the largest cluster encompassing the most keywords, followed by #1, "model," and #2, "synaptic plasticity," among others. Color should be used for any figures in print.

To gain a more intuitive understanding of the current research topics related to dynamic balance and brain structure, we conducted clustering and summarization of these keywords (see Fig. 5). The clustering process yielded a Q value of 0.4034, indicating that clustering is appropriate and meaningful. Fig. 5 illustrates the cluster view of keywords in the literature exploring the relationship between human dynamic balance and brain function. This visualization, generated using the CiteSpace algorithm, provides insights into the structural characteristics among clusters formed by grouping similar keywords. This high-level perspective enhances comprehensive understanding of the literature. The cluster view highlights key nodes and significant connections, with nodes exhibiting high mediating centrality potentially indicating emerging trends in the field.

### 3.5 Co-occurring institution analysis

**Fig. 6:**
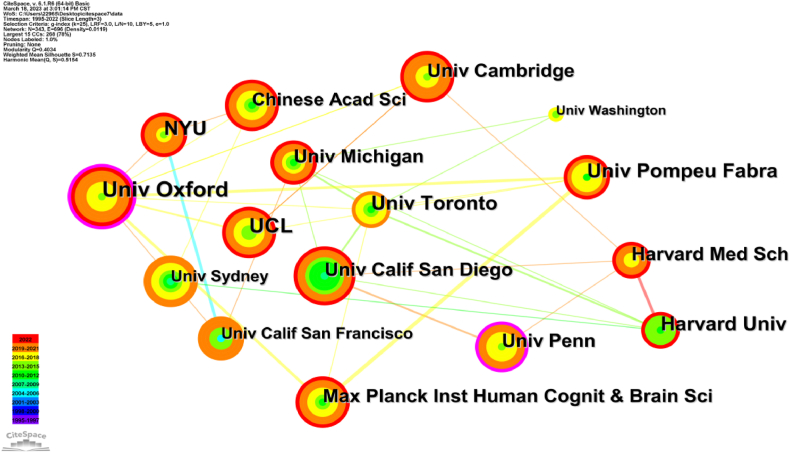
Institution cooperative network map. Node size represents the volume of related literature published, and the timeline is displayed in the lower left section. Color should be used for any figures in print.

Fig. 6 illustrates the analysis diagram of institutional cooperation networks in the literature exploring the relationship between human dynamic balance and brain function. It highlights the top 15 organizations, their importance indicators, and network attributes. Among the 343 closely observed institutions within this research area, there are 696 links between them (see Fig. 6). The University of Oxford stands out with a higher centrality, as indicated by the purple circle. Both the University of Oxford and the University of Pennsylvania have a long period of publication. The top five institutions are all universities. The University of Oxford leads with 35 published papers, followed by the University of Sydney and New York University, both with 30 papers (see Table 4). Table 4 shows the top five institutions, all of which are universities and have strong centrality.

**Table 4:** The top 5 most frequently used Institution.

Rank	Frequency	Centrality	Year	Institution
1	35	0.12	2007	Oxford University
2	30	0.05	2001	Sydney University
3	30	0.09	2001	New York University
4	28	0.06	2010	London University
5	24	0.06	2010	Michigan University

### 3.6 Co-occurring country analysis

**Fig. 7:**
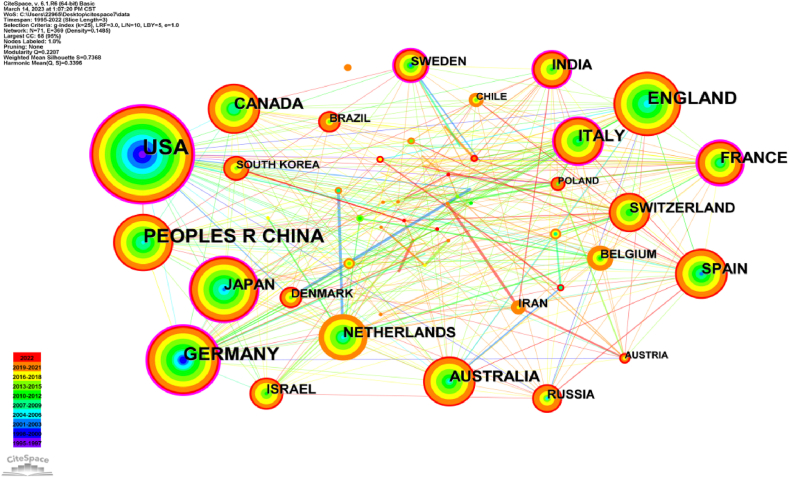
Co-occurrence map of Country. The size of the node circle in the figure corresponds to the volume of related literature published by each country. The timeline, indicated in the lower left corner, showcases the publication year distribution, with earlier years represented by red and later years by purple. Color should be used for any figures in print.

Fig. 7 presents the analysis diagram of the national cooperation networks in the literature exploring the relationship between human dynamic balance and brain function. It illustrates 71 nodes and 369 links, revealing the national contributions and collaboration in this field. Human dynamic balance and related aspects of brain structure, are a global concern, reflected in the 1533 articles published by researchers from 71 countries.

Table 5 shows the top 5 countries based on the number of publications. The United States emerges as the major contributor, accounting for one-third of the total articles (565) and publishing earlier than any other country. It is followed by People's Republic of China (245) and Germany (195). England and France followed closely behind, with over 100 articles each, while the remaining countries have comparatively lower publication numbers. Among the top five countries, the United States and France published the earliest relevant articles on dynamic balance and brain structure around 1995. Notably, all five countries are developed or developing nations with high economic and technological levels, fostering the advancement and development of brain balance technology.

**Table 5:** Top 5 countries in number of publications related to brain balance.

Rank	Publications	Centrality	Year	Countries
1	565	0.36	1995	United States
2	245	0.08	2005	People's Republic of China
3	195	0.29	1996	Germany
4	167	0.08	1998	England
5	103	0.22	1995	France

In Fig. 7, nodes represent countries, and the purple ring represents the centrality of literature. Strong centrality is observed in the United States (0.36), Germany (0.29), France (0.22), Japan (0.14), Italy (0.13), India (0.13), and Sweden (0.13). The United States demonstrates a prominent position in terms of both publication numbers and importance.

### 3.7 Co-occurring Reference analysis

**Fig. 8:**
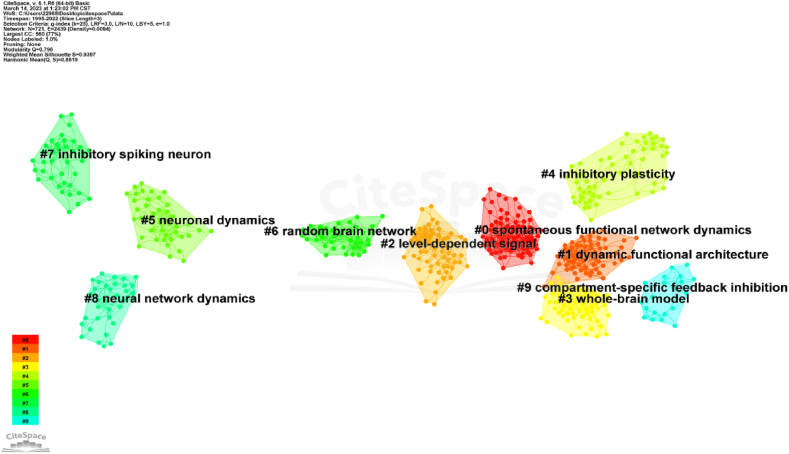
Cluster map of Reference. The color of each cluster block represents the year in which the word relationship first emerged within the cluster. Overlapping blocks suggest the extension and ongoing development of previous fields of research. Color should be used for any figures in print.

The collaborative network cluster map comprises 721 nodes and 2439 links, as illustrated in Fig. 8. The figure presents 10 clusters labeled from #0 to #9, with lower sequence numbers indicating larger cluster sizes and a greater number of associated keywords. Cluster #0, “spontaneous functional network dynamics”, stands out as one of the largest clusters. A significant article within this cluster is by R. Metthew Hutchison (38) [32], which discusses dynamic functional connectivity and the brain's ability to integrate, coordinate, and respond to various internal and external stimuli. This article underscores the advancements in noninvasive measurement of brain activity using functional magnetic resonance imaging (fMRI) and reviews recent research findings, methodologies, considerations, neural and behavioral correlations, as well as future directions.

Another influential review tied for first place within this cluster is Preti et al.'s (2017) work on the dynamic functional connectome. This comprehensive review emphasizes the investigation of functional interactions between different brain regions, previously believed to have a static nature over extended periods. However, recent studies have revealed the dynamic nature of the functional connectome, demonstrating meaningful changes in typical resting-state fMRI experiments alongside the correlated patterns of spontaneous fMRI signal fluctuations.

Table 6 presents details of one of the most frequently cited references within the top ten texts. The table includes information such as citation counts, title, author, year, and journal. The paper by Hutchison et al. (2013) is the most cited reference on resting brain networks, with 475 citations since its publication in 2014. The second most cited paper, published in 2017, focuses on fMRI signals and has accumulated 284 citations. Notably, these articles are closely associated with fMRI techniques [33], the cerebral cortex [34], and cerebral networks [35].

**Table 6:** Top 10 most cited articles.

Rank	Citation counts	Tile	Author	Year	Journals
1	475	Time-resolved resting-state brain networks	Zalesky, A et al.	2014	Proceedings of the National Academy of Sciences of the United States of America (PNAS)
2	284	On the Stability of BOLD fMRI Correlations	Laumann, TO et al.	2017	Cerebral Cortex
3	226	Evaluating dynamic bivariate correlations in resting-state fMRI: A comparison study and a new approach	Lindquist, MA et al.	2014	Neuroimage
4	154	Human brain networks function in connectome-specific harmonic waves	Atasoy, S et al.	2016	Nature
5	152	Network-Level Structure-Function Relationships in Human Neocortex	Misic, B et al.	2016	Cerebral Cortex
6	134	The effect of epoch length on estimated EEG functional connectivity and brain network organisation	Fraschini, M et al.	2016	Journal of Neural Engineering
7	131	Interpreting temporal fluctuations in resting-state functional connectivity MRI	Liegeois, R et al.	2017	Neuroimage
8	122	Network Structure Shapes Spontaneous Functional Connectivity Dynamics	Shen, K et al.	2015	Journal of Neuroscience
9	110	Interactions between the default network and dorsal attention network vary across default subsystems, time, and cognitive states	Dixon, ML et al.	2017	Neuroimage
10	109	Comparing test-retest reliability of dynamic functional connectivity methods	Choe, AS et al.	2017	Neuroimage

**Fig. 9:**
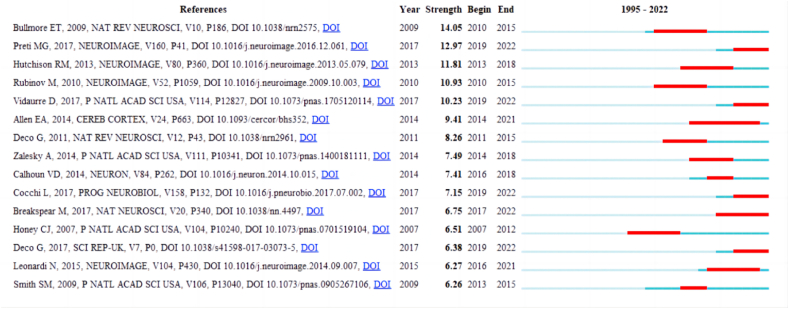
Top 15 References with the Strongest Citation Bursts. These references with the strongest citations all exhibit significant explosive intensity, and the start to end times are indicated by red terms. Color should be used for any figures in print.

The growing research interest in human dynamic balance and the structural and functional connectivity of the brain is evident from the increasing number of citations in publications. Fig. 9 illustrates the top 15 citations with the most significant bursts. The earliest article with the highest burst, with an intensity of 14.05, was published in 2010 and focuses on the analysis of complex brain networks using graph theory [36].

This article reviewed studies of complex brain networks under various experimental modes, including structural and functional MRI, diffusion tensor imaging, magnetoencephalogram, and electroencephalogram. It also provides a brief overview of the fundamental principles of graph theory. Furthermore, the article addresses the technical challenges and key issues that must be addressed for the future advancement of this rapidly growing field.

## 4 Discussion

This study employs CiteSpace, a bibliometrics and visual analysis tool, to examine the research literature on human dynamic balance and brain function as well as connectivity within the Web of Science database. 1533 publications from the period 1995 to 2022 was obtained, providing a comprehensive overview of the research landscape, including its hotspots, frontiers, and trends.

The findings of this study indicate a consistent growth in publications related to human dynamic balance and brain structural and functional connectivity in recent years. Notably, the largest increase occurred between 2020 and 2021 (see Fig. 1). Among the journals analyzed, the *Proceedings of the National Academy of Sciences* (PNAS) published the most papers (1,053) (see Table 1). Additionally, noteworthy contributions have been made by authors such as Gustavo Deco and research teams affiliated with University of Oxford.

The visualization analysis of authors (see Fig. 2) and their institutions (see Fig. 6) provides valuable insights for identifying potential collaborators and teams for future researchers. The authors conducted randomized controlled trials [37] and systematic reviews [38] to compare the effects of regular rehabilitation training and personalized balance training on patients. The results indicated that the therapeutic effect of specialized balance training was equivalent to routine rehabilitation, with high levels of patients and family satisfaction [39].

Physical exercise has been demonstrated to induce structural plasticity in the human brain and enhance cognitive function [40]. Balance training, specifically, has shown to increase cortical thickness in the visual and vestibular cortex areas [41]. While balance training has been shown to be effective in counteracting the worsening of gait disorders, its impact on brain activity during postural tasks in older adults appears to be less pronounced. Studies assessing postural stability and brain activity using fMRI during motor imagination and combined motor observation have revealed that balance training can reverse age-related cortical overactivation and lead to changes in the control of upright postures, resembling those observed in young individuals [42].

The institutional map highlights extensive collaboration among various universities, particularly the University of Oxford（35 publications）, the University of Sydney（30 publications）, and New York University (30 publications), demonstrating a strong inclination of universities towards research in this field (see Table 4 or Fig. 6). Additionally, the geographical distribution of research institutions reveals a concentration in city center, indicating better research conditions and access to resources, including talent and funding, etc.

The analysis of keywords (see Fig. 3, Fig. 4) reveals significant patterns in research interest and trends. The term “brain” emerges as the most central keyword, indicating its extensive attention within the academic community. It exhibits a pronounced burst of activity, particularly during the period from 1996 to 2009, suggesting a deep level of research interest. Other frequently occurring and highly central keywords include “neural network”, “functional connectivity”, “dynamics”, “disease” and “model”. This suggests that with advancements in technology, there will be an increasing focus on network models [43].

Gait and balance disorders are prominent clinical manifestations of various neurodegenerative diseases [44]. Keywords such as “FMRI” and “EEG” emerge prominently, indicating the application of medical devices in this research field. For instance, in the context of Parkinson's disease, brain functional imaging technology can be employed to analyze the characteristics and patterns of brain functional changes related to gait disorders [45]. Resting state functional magnetic resonance imaging (RS-fMRI) has become a valuable tool for studying the connective structure of mental health disorders [46]. Most of these studies rely on measures of overall mean functional connectivity, based on temporal correlations between BOLD time series of different brain regions. However, the neurophysiological processes underlying non-invasive brain activity measurements are not fully understood. To address this, a connectome-based brain network model has been developed, which combines individual structure and function data with neural population dynamics to support multi-scale neurophysiological inference [47]. Simulated populations are linked together by structural connections and, in a novel way, driven by electroencephalogram (EEG) source activity. This has significant clinical implications for understanding the neuropathophysiological mechanisms, guiding treatment strategies, and evaluating treatment outcomes.

Cluster analysis conducted by CiteSpace aims to identify similarities among different data sources and classify them into different clusters. However, this process may lead to some overlap in clustering results. By examining the clustering words and the keywords contained within each cluster (see Fig. 5), we can analyze and speculate on the 10 clustering groups. These clusters can be broadly categorized into the following areas: (1) functional connection, (2) model, (3) Alzheimer's disease, (4) system, and so on. One group of keywords focuses on the functional connectivity of the brain and the development of various models, while another group delves into the in-depth study of neurodegenerative diseases. By conducting a burst analysis of keywords (see Fig. 4), one can obtain a general overview of the research field on dynamic balance and brain functional structure, research development, and future research trends.

However, there are certain imitations to this research: (1) The analysis using CiteSpace may be subject to controversy when compared to expert opinions in the field; (2) Our current study is confined to research within the Web of Science database. Future directions include integrating data from additional sources, conducting qualitative assessments, seeking expert opinion, and incorporating case studies to enhance depth and comprehensiveness of our understanding of the field; (3) The lack of standardization in setting time partition, threshold values, and cutting modes lacks within the software required repeated exploration to approximate accurate settings, which may have impacted the precision of the results; (4) Clustering the included data may result in overlapping content from different categories, necessitating a cautious interpretation of the research findings in conjunction with clinical practice.

## 5 Conclusions

To summarize, we used CiteSpace for co-citation analysis to visualize the literature network related to dynamic balance and brain structure as well as functional connectivity. This exploration helped identify emerging trends in this research field, including author and institutional collaboration networks, hot topics, and research development directions. Most of the research on dynamic balance and brain structure has been carried out in developed countries, fostering a cooperative network among authors.

Currently, the primary research focus of dynamic balance and brain structure as well as functional connectivity lies in neurodegenerative diseases. Future research may shift towards the development of brain function techniques such as deep brain stimulation, which will increasingly contribute to the rehabilitation of gait disorders. Additionally, brain injury-related network connectivity changes represent a significant research direction in this field. In the future, the research on dynamic balance and brain structure as well as functional connectivity will emphasize the cooperation between various brain networks to enhance gait balance of patients and improve their quality of life. As science and technology continue to progress, our understanding and advancements in dynamic balance and brain functional connectivity are bound to evolve further.

## Funding

This research was supported by the 10.13039/501100003392Natural Science Foundation of Fujian Province [Grant Number 2022J01890]; 10.13039/501100001809National Natural Science Foundation of China [Grant Number 12002177]; Science and 10.13039/100006180Technology Development Programme 2022 of Chinese Association of Rehabilitation Medicine [Grant Number KFKT-2022-016]; Quanzhou Science Technology Project [Grant Number 2022FX7]; and Project of Philosophy and Social Sciences of Zhejiang Province [Grant Number 20NDQN276YB]. The study was also supported by K.C. Wong Magna Fund in 10.13039/501100004387Ningbo University.

## Data availability statement

The data that support the findings of this study are available on request from the corresponding author.

## CRediT authorship contribution statement

**Mengjiao Liu:** Writing - original draft, Formal analysis, Data curation. **Jian He:** Writing - review & editing, Supervision. **Dongwei Liu:** Writing - original draft, Methodology, Funding acquisition. **Meijin Hou:** Writing - review & editing, Funding acquisition, Formal analysis. **Ye Ma:** Writing - review & editing, Supervision, Methodology, Funding acquisition, Formal analysis, Conceptualization.

## Declaration of competing interest

The authors declare that they have no known competing financial interests or personal relationships that could have appeared to influence the work reported in this paper.
